# The Effect of Multi-Session Prefrontal Cortical Stimulation on Aggression: A Randomized, Double-Blind, Parallel-Group Trial

**DOI:** 10.3390/life13081729

**Published:** 2023-08-11

**Authors:** Olivia Choy, Gary Tan, Yen Cong Wong

**Affiliations:** 1 Department of Psychology, Nanyang Technological University, 48 Nanyang Avenue, Singapore 639818, Singapore; gary0018@e.ntu.edu.sg; 2 Centre for Research on Successful Ageing, School of Social Sciences, Singapore Management University, 10 Canning Rise, Level 5, Singapore 179873, Singapore; wong1139@e.ntu.edu.sg

**Keywords:** aggression, prefrontal, non-invasive brain stimulation, transcranial direct current stimulation, violence

## Abstract

Findings from brain imaging studies investigating the neural underpinnings of antisocial behavior have implicated the prefrontal cortex in the regulation of aggressive reactions. However, relatively few studies have examined the role of the prefrontal cortex on aggression in an experimental way. This study examines whether upregulating the prefrontal cortex using repeated transcranial direct current stimulation (tDCS) reduces aggressive behavior. In a double-blind, parallel-group, randomized controlled trial, 88 healthy adults (42 males, 46 females) were assigned to one session of anodal tDCS over the ventromedial prefrontal cortex (n = 47) or sham stimulation (n = 41) per day for three consecutive days and assessed using a behavioral measure of aggression. Levels of aggressive responses post-intervention did not significantly differ between the active and sham stimulation groups. However, a significant interaction effect between the stimulation group and gender was observed, whereby males, but not females, exhibited reduced aggression after prefrontal stimulation. To the authors’ knowledge, this is the first study to examine the effect of multi-session prefrontal tDCS on aggressive behavior in healthy adults. Results highlight that there are differences in responsivity to tDCS in modifying aggressive behavior.

## 1. Introduction

The costs of aggression to society, which span the areas of health, social care, and criminal justice, are enormous. Worldwide, the annual cost of violence is estimated to be $9.4 trillion [1]. This is particularly notable for males. An examination of the long-term cumulative cost of behavior problems in a birth cohort showed that relative to individuals with fewer conduct problems, the costs for those with high levels of conduct problems were doubled for girls and greater by six-fold for boys [2]. This highlights a clear need to better understand the etiology of aggression and to identify ways to manage it.

In addition to social and environmental factors, there is increasing acknowledgment that neurobiological influences matter in aggression and violence. Animal research many decades ago indicated the involvement of the brain in aggressive behavior and demonstrated that such behavior can be altered by stimulating the brain [3]. In humans, findings from functional and structural neuroimaging studies provide support for a neural circuit model of aggressive behavior that implicates interconnected regions of the brain that play a role in emotional processing and cognitive control [4]. These include brain areas that are located in the prefrontal cortex [5]. In particular, neuroimaging data suggest that reactive aggression resulting from provocation is modulated by the ventromedial prefrontal cortex (vmPFC) [6]. Structurally, in healthy adults, reduced gray matter volume and density in the vmPFC were associated with increased physical aggression [7]. Functionally, deactivation in the vmPFC occurred when healthy individuals were engaged in imagined scenarios of physical aggression [8]. The negative relationship between vmPFC activity and aggressive behavior is further supported by research on patients with damage to this brain area. Neurological studies show that lesions to the vmPFC are associated with impairments in impulse control, understanding others’ mental states, and real-world decision-making, as well as increases in aggressive behavior [9,10,11,12]. 

However, evidence of the involvement of the prefrontal cortex in aggressive behavior has largely been correlational in nature. To buttress these conclusions and advance our knowledge about the neural basis of aggression, the role of the prefrontal cortex on aggressive behavior can be examined in an experimental way. One way to investigate relationships between the brain and behavior is by using transcranial direct current stimulation (tDCS). tDCS is a non-invasive brain stimulation technique that can be used to transiently modulate cortical function. It involves delivering a direct, continuous, low-intensity electrical current to cortical areas between anodal and cathodal electrodes [13]. Stimulation applied via the anode, an electrode with a positive charge, generally acts to enhance cortical excitability, while stimulation delivered through the cathode, a negatively-charged electrode, is thought to decrease excitability. However, the conventional relationship between current flow polarity and cortical excitability can be dependent on specific neurostimulation parameters, including where electrodes are positioned. For example, the anodal-excitation and cathodal-inhibition effects were found to be less robust outside of the motor cortex [14]. This is particularly the case for cathodal stimulation. Meta-analytic evidence revealed that anodal tDCS over non-motor regions mainly resulted in the expected excitatory effect, but the inhibitory effects of cathodal stimulation were less consistently observed [14]. The effects of tDCS can also vary depending on the timing of stimulation in relation to task performance. Prior studies show that tDCS-induced shifts in neuronal membrane potentials that increase the likelihood of neurons firing preferentially affect neuronal networks that are already engaged in activity [15] and that prefrontal tDCS yielded larger effects when a behavioral outcome was assessed after, rather than during stimulation [16]. It is also documented that stimulation intensity and duration can influence tDCS effects, with the most common tDCS protocols involving electrodes ranging from 25 to 35 cm^2^ and currents of 1 to 2 mA for 20 to 40 min [13]. The low current density on the scalp allows for minimal sensations by participants, so that they can be blinded in randomized controlled trials of real and sham stimulation. 

Recent studies employing tDCS have provided some initial evidence that it can alter aggressive behavior by directly changing brain activity. They involve using anodal stimulation to influence neuronal excitability on frontal sites and have largely examined behavior in community-dwelling samples of healthy subjects. One such study aimed to enhance vmPFC activity. It found that one session of anodal stimulation over the vmPFC resulted in reduced aggressive behavior and that this effect was influenced by the order of stimulation condition [17]. Anodal tDCS over the right ventrolateral prefrontal cortex (vlPFC) has also been shown to reduce aggressive responses in a behavioral lab task [18]. This reduction in aggression due to tDCS was similarly observed when participants were socially excluded [19] and after they were exposed to violent video games [20]. In another study of prefrontal cortical stimulation, responses to hypothetical vignettes showed that behavioral intentions to commit physical and sexual assault were lower in individuals who received anodal tDCS over the dorsolateral prefrontal cortex (dlPFC) compared to those who received sham stimulation [21]. However, mixed findings also exist. Some randomized controlled trials revealed no significant effects of bilateral prefrontal tDCS on aggressive behavior [21,22,23]. One study additionally found that participants who received tDCS to increase activity in the left frontal cortex exhibited more aggressive responses when they were angry [24]. 

As these studies have relied on the use of a single session of anodal prefrontal cortical stimulation, one question that has arisen from the observed null effects on aggression is whether one session of tDCS may be enough to elicit behavioral change. It has been suggested that multiple stimulation sessions may be needed to optimize the effects of tDCS. The notion that effects may be more potent with repeated exposure to stimulation is supported by evidence that compared with single-session tDCS protocols, multi-session protocols show a larger effect size for altering behavioral and cognitive responses related to drug use, which has ties to antisocial and aggressive behavior [25]. To date, only two studies have examined the effect of multiple sessions of tDCS on aggression. Both studies have been conducted in correctional and forensic samples. In males incarcerated for violent offenses, three sessions of bilateral anodal stimulation over the dlPFC on consecutive days yielded reductions in self-reported physical and verbal aggression [26]. A second study investigated the effect of high-definition tDCS over the vmPFC on aggression in forensic substance-dependent patients [27]. It found behavioral aggression to be significantly reduced post-intervention in forensic patients who received stimulation twice a day on five consecutive days, but not for patients in the sham group. Despite the small body of research, the extant preliminary experimental evidence shows the effectiveness of repeated prefrontal stimulation in modifying aggressive behavior. 

The inconsistent findings from studies on tDCS and aggression have also stemmed from gender differences. Interaction effects between gender and stimulation condition have been documented. For example, one study found that increasing activity in the right prefrontal cortex reduced proactive aggression in males, but not in females [28]. In another study, although males exhibited greater aggressive behavior than females after sham stimulation, the difference in aggression levels between males and females was significantly reduced after anodal tDCS over the right and left vlPFC [29]. These results showing that females who received prefrontal cortical stimulation were as aggressive as males suggest that there may be differences in the way males and females respond to the effects of tDCS on aggressive behavior.

### Current Study

This study aims to build on the nascent body of experimental research on the prefrontal-aggression relationship by addressing two key issues. First, although the vmPFC has been suggested to be a target for tDCS intervention, only two prior studies have investigated the effect of stimulation over this brain region on aggression [17,27]. Second, although three sessions of prefrontal tDCS reduced aggression in male violent offenders [26], the question of whether repeated sessions of tDCS can yield changes in aggressive behavior in a non-institutionalized sample remains unexamined. Thus, we test the hypothesis that repeated sessions of anodal stimulation over the vmPFC, which may be associated with a cumulative tDCS effect, reduces aggressive behavior in a community sample of healthy adults.

This is conducted by using a behavioral measure of aggression, the Point Subtraction Aggression Paradigm (PSAP), which has been suggested to have increased ecological validity [30], yet has not been adopted in prior studies using conventional tDCS methods. This behavioral task allows participants to respond either aggressively or non-aggressively, and aggressive choices are made at a cost to participants, which are akin to real-world situations. This lowers the likelihood of overestimating aggression levels that would occur outside of the laboratory. Additionally, gender differences in tDCS effects are examined. 

## 2. Methods

### 2.1. Participants 

A total of 123 healthy adults (≥21 years of age) were recruited between November 2019 and December 2020. Participants were excluded if they had contraindications to brain stimulation, including metallic implants near the electrode sites. Other exclusion criteria included having unstable medical conditions, neurological, cardiovascular, or psychiatric illness, consumption of anti-convulsant, anti-psychotic, or sedative/hypnotic medications or anti-depressants, participation in another non-invasive brain stimulation study on the same day, and history of adverse reactions to tDCS. Written informed consent was obtained from all participants. 

This study recruited a larger sample of healthy adults than in prior studies on tDCS and aggression hitherto. A review of all existing experimental studies on non-invasive brain stimulation and aggression showed that across 10 studies that employed tDCS, there was, on average, a total of 55 participants per study, with only one study recruiting more than 80 subjects [31]. Furthermore, following power analysis used in prior research to examine the reproducibility of tDCS effects, to achieve 80% power for detecting an effect size of *d* = 0.6, assuming one-tailed testing and alpha error probability of α = 0.05, a total sample size of at least 72 participants was needed [32]. 

### 2.2. Trial Design 

This study consisted of a double-blind, randomized trial in which an anodal tDCS intervention was compared with a sham, placebo condition. Randomization was carried out using a simple randomization method prior to the recruitment of participants by a researcher who did not administer the tDCS. Immediately after baseline assessments were conducted, the first stimulation session was run. One stimulation session was administered each day during visits to the study site at the same time on three consecutive days. Participants were assigned to the same stimulation protocol on all three days, but were not informed about this. Questionnaires used to measure baseline variables and post-stimulation outcomes, as well as laboratory tasks during and after the stimulation period, were administered in the same order for all participants. This study was approved by the Institutional Review Board of Nanyang Technological University. The trial protocol was registered at ClinicalTrials.gov (NCT04204759).

### 2.3. tDCS 

tDCS was administered by trained study personnel using a constant-current stimulator (Starstim Neuroelectrics, Barcelona, Spain) and the associated NIC2 software (version 2.0.6). Head measurements were first performed on participants to select the appropriate size of the neoprene EEG cap in which electrodes were placed. Based on previously established procedures for vmPFC stimulation [33], one anodal electrode was placed over the Fpz according to the International 10–20 EEG system. A constant current of 2 mA was applied for 20 min through a 25 cm^2^ saline-soaked sponge electrode. The reference electrode (35 cm^2^) was placed over the Oz position. Thus, both electrodes were at midline points. The placement of the return electrode in this position has been suggested to reduce the risk that the cathode indirectly and unintentionally confounds the behavior of the participant [31]. Electrode impedance checks were made before and during stimulation. Excessive impedance between the electrodes resulted in the discontinuation of a stimulation session. Sensations experienced during tDCS were recorded using a self-reported questionnaire after each stimulation session [34].

Following standard tDCS protocol, stimulation began after a 30-s ramp-up period. The current ramped down over the last 30 s. During the stimulation sessions, all participants performed two cognitive tasks that are known to engage the vmPFC, the Iowa Gambling Task [35,36], followed by the Probabilistic Reversal Learning Task [37,38]. 

Multiple steps were taken to maximize the validity of the results. First, participants and experimenters were blind to assignments to real and sham stimulation conditions. Both groups had an identical setup. All participants received the same electrode placement and ramp-up/down times. However, stimulation for the sham control group was discontinued after 30 s. This has proven to be effective for blinding as participants habituate to the sensation of stimulation within seconds of current initiation [39]. The tDCS protocols were preconfigured by a researcher who was not involved in the testing sessions and were administered in blind mode, concealing the stimulation parameters for the experimenter. Second, all participants were kept blind to the objective of this study. Third, baseline and outcome measures were obtained with research staff in an adjacent room in order to reduce the risk of producing biased responses in their presence. 

### 2.4. Aggressive Behavior

The PSAP was used as a measure of behavioral aggression [40]. The paradigm was delivered using the Inquisit Lab software version 5.0 (Millisecond, Seattle, WA, USA). It was administered once after the third stimulation session. In this task, participants were paired with a fictitious partner and tasked to win as many points as possible. They were also informed that points could periodically be stolen from them by their opponent. Participants had the option of responding to the provocation in one of three ways. They could (a) earn one point by pressing a key on the keyboard 100 consecutive times, (b) steal a point from their opponent by pressing a second key 10 consecutive times, and (c) protect their points from being stolen for a limited time by pressing a third key 10 consecutive times. Choosing options (b) and (c) initiated a 250-s interval during which no additional points were subtracted from the participants’ point counters. Thus, each participant could experience up to 25 provocations. Although points were subtracted from their point counter when they were stolen by the opponent, participants were informed that points that they stole from their opponent would not be added to their counter. In this way, stealing a point inflicted a negative outcome on the opponent, but it did not financially benefit the participant and detracted from the possibility of earning a point. 

Aggressive responding served as the outcome variable in this study. It was defined as the number of option (b) presses divided by the total number of key presses times the number of provocations received, scaled by 1000 (i.e., [1000 × number of option (b) presses]/[number of total key presses × number of provocations]). This index was used in order to adjust for individual differences in the rate of button presses and received provocations [41].

### 2.5. Covariates

Baseline measures of aggression, psychopathy, and offending behavior were examined as possible covariates. Other variables that could influence the outcome variable were considered, such as impulsivity, emotion regulation, self-control, and social adversity. Before the intervention, levels of aggression were assessed using the Reactive-Proactive Aggression Questionnaire [42]. Psychopathic traits were evaluated using the short form of the Self-Report of Psychopathy-III Questionnaire [43]. As a recommended method to summarize the extent of individual criminality with high reliability and validity [44], a variety score of crimes committed throughout the lifetime was calculated from participants’ reports of the number of times they had engaged in 18 criminal and delinquent acts ranging from white-collar to blue-collar offenses (e.g., fraud, shoplifting, illegal drug use, driving under the influence of alcohol or drugs). Additionally, scores from the lack of premeditation and sensation-seeking subscales of the short-form version of the UPPS-P Impulsivity Scale were obtained [45]. Individual differences in two emotion regulation strategies, cognitive reappraisal and expressive suppression, were measured using the self-reported Emotion Regulation Questionnaire [46], and self-control was assessed using the Brief Self-Control Scale [47]. Social adversity was measured based on self-reported responses to 13 demographic items. Indicators used to calculate a social adversity index included mother’s low education, father’s low education, parental separation or divorce, placement in a foster home, hospital, or other institution during childhood, having 5 or more siblings, born to a teenage mother, a ratio of people per room of 1.0 and above, brought up in public housing, parents’ use of welfare or food stamps from the government, father or mother had been arrested, father or mother has had problems with alcohol or drugs, father or mother has had physical illness, such as heart or lung problems, and father or mother has had mental illness, such as alcoholism, major depression, schizophrenia, or anxiety. 

### 2.6. Statistical Analyses

Analysis of variance (ANOVA) was used to test group differences in aggressive behavior. Analysis of gender differences was conducted using a 2 (stimulation group) × 2 (gender) between-subjects ANOVA. Following recommendations, variables measured at baseline, prior to real or sham stimulation, that were found to be associated with the outcome were controlled for in the analysis, while those with observed imbalances at baseline were not [48,49]. To determine this, associations between aggressive responses and baseline characteristics were assessed. Pearson correlations were used for continuous baseline variables, and *t*-tests were employed for dichotomous demographic variables. 

A per-protocol analysis was performed as the primary analysis. This allowed for the assessment of whether anodal prefrontal tDCS had an effect on aggressive behavior. The consideration of this study as an explanatory trial rather than a management trial is in line with recommendations that an intervention should be established to work in a controlled situation before evaluating treatment effectiveness in a practical setting and under usual clinical circumstances [50]. Nevertheless, intention-to-treat analysis was conducted for the purpose of sensitivity testing, in which data from all subjects who were randomized to a stimulation group were analyzed. Data were imputed for participants with missing data on the outcome variable by replacing missing values with the mean of the other group. This approach has been shown to be associated with no significant loss of power and to be more robust regarding type I error for moderate drop-out rates [51].

Assessments of adherence to blinding procedures were made after each of the three stimulation sessions that participants underwent. Based on the participants’ and experimenters’ guesses about group assignments at the end of the experimental sessions, James’ and Bang’s blinding indices were calculated using STATA version 16.0 (Stata Corp., College Station, TX, USA, 2019) [52,53]. All other statistical analyses were conducted using SPSS version 29.0 (IBM Corp., Armonk, NY, USA, 2022). 

## 3. Results

### 3.1. Participant Flow and Recruitment

**Figure 1:**
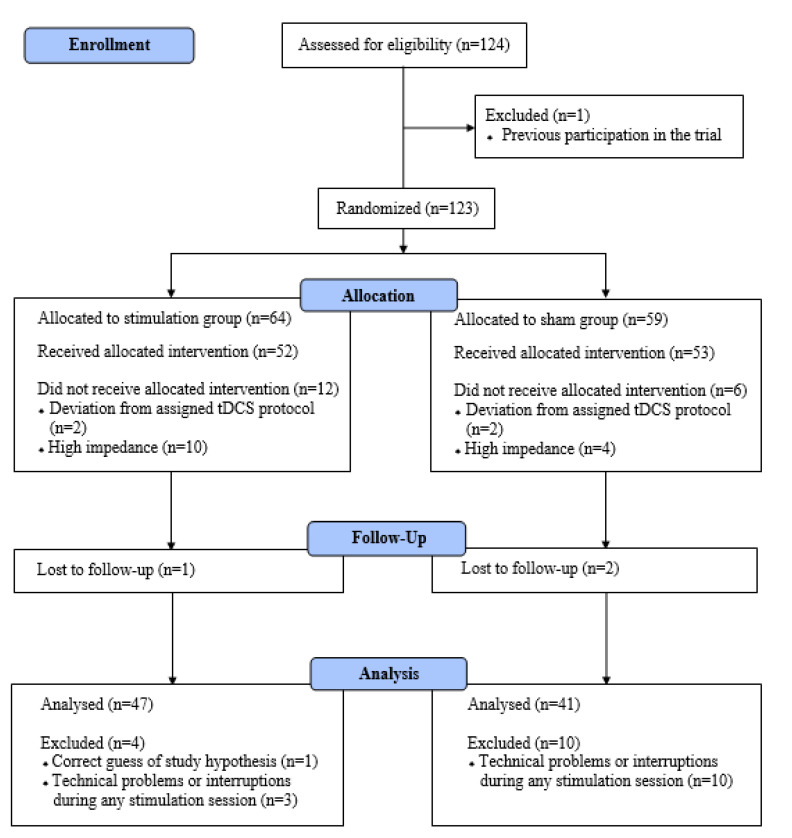
CONSORT flowchart of the screening and enrollment of study participants.

Data were analyzed on a total sample of 88. The Consolidated Standards of Reporting Trials (CONSORT) flow chart presents details for loss (Figure 1). Besides an observed difference in sensation-seeking, no other evidence of selection bias was found (Table 1). No significant differences were observed in gender, age, ethnicity, or other baseline variables between participants who were included and excluded in the analyses (*p* > 0.05). 

### 3.2. Baseline Characteristics

The active and sham stimulation groups were generally well balanced with respect to gender, ethnicity, antisocial and aggressive behavior, impulsivity, emotion regulation, self-control, and social adversity. No significant differences in these demographic variables and baseline characteristics were found between participants from both groups (Table 2). The sham group exhibited a greater lack of premeditation compared with participants in the active stimulation group, but this difference did not meet statistical significance. Only age significantly differed between the stimulation groups, with the active tDCS group being older on average. An examination of gender differences in variables measured at baseline showed that males had significantly higher psychopathy and offending scores than females (Table 2). On average, they also were older and experienced more social adversity than females, but these gender differences were not statistically significant.

### 3.3. Adherence and Tolerance to Protocol

Analysis of blinding effectiveness showed that across sessions, James’ blinding indices ranged from 0.57 to 0.84, and Bang’s blinding indices ranged from −0.29 to 0.51 (Table 3). As James’ and Bang’s indices were greater than 0.5 and did not approach 1 or −1, respectively, both participants and experimenters were considered to be blinded successfully on average [52,53]. There were no incidences of withdrawal from or discontinuation of a stimulation session due to a reported adverse event. Across all of the stimulation sessions conducted, the most common sensation was itchiness, which occurred in 68.84% of the sessions. Lightheadedness, warmth, pinching, pain, burning sensation, iron taste, and fatigue were reported in 26.35%, 28.32%, 34.84%, 33.14%, 24.36%, 6.80%, and 39.94% of the stimulation sessions, respectively. These reports are consistent with meta-analytic findings that serious adverse effects of tDCS within standard protocols are rare [54]. 

### 3.4. Associations between Baseline Characteristics and Aggressive Behavior

Bivariate associations between the hypothesized covariates and outcome measures were examined to determine prognostic covariates. The correlational analysis showed that there were no statistically significant associations between aggressive responding on the PSAP and age (*r* = −0.02, *p* = 0.88), baseline aggression (*r* = 0.12, *p* = 0.28), psychopathy (*r* = 0.15, *p* = 0.16), variety of offending (*r* = 0.13, *p* = 0.23), lack of premeditation (*r* = 0.09, *p* = 0.43), sensation-seeking (*r* = 0.05, *p* = 0.67), cognitive appraisal (*r* = 0.07, *p* = 0.54), self-control (*r* = −0.09, *p* = 0.40), social adversity (*r* = 0.05, *p* = 0.66), or expressive suppression (*r* = 0.20, *p =* 0.07), although a trend was observed for the latter. Males exhibited higher levels of aggression compared to females, but this gender difference was not statistically significant, *t*(86) = −1.81, *p* = 0.07. Chinese participants exhibited a non-significantly higher level of aggressive responses relative to non-Chinese participants, *t*(86) = −1.92, *p* = 0.06.

### 3.5. tDCS Effects on Aggressive Behavior

A one-way ANOVA revealed that aggressive behavior after multi-session tDCS did not significantly differ between the active (mean = 41.62, SD = 42.65) and sham stimulation (mean = 46.60, SD = 41.77) groups, *F* (1, 86) = 0.30, *p* = 0.58. Further analysis showed that there was no significant interaction effect between the stimulation group and ethnicity, *F* (1, 84) = 0.71, *p* = 0.40, η_p_^2^ = 0.01. However, a significant interaction effect was observed between the stimulation group and gender, *F* (1, 84) = 5.02, *p* = 0.03, η_p_^2^ = 0.06. 

**Figure 2:**
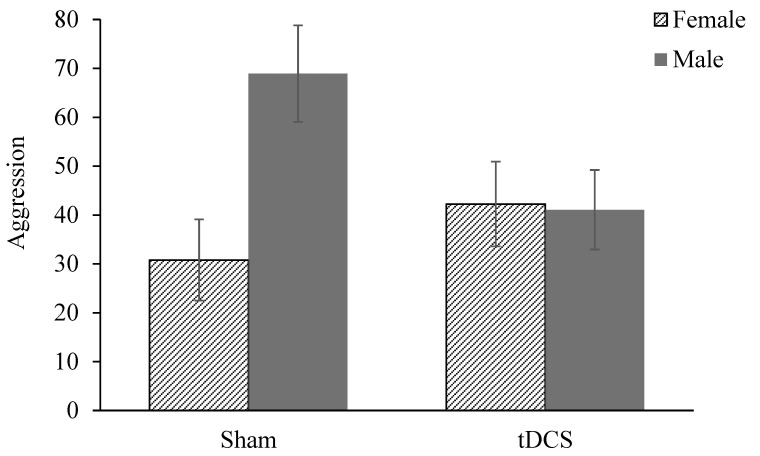
Aggressive responding score in the PSAP by stimulation group and gender.

On average, males exhibited more aggressive responding (mean = 52.35, SD = 45.14) compared to females (mean = 36.27, SD = 37.93; *p* = 0.07). Additional analysis conducted to examine gender effects revealed that in males, there was a main effect of stimulation on aggression, *F* (1, 40) = 4.15, *p* = 0.048. Compared with males who received sham stimulation, those who received anodal stimulation over the prefrontal cortex engaged in lower levels of aggression after tDCS (Figure 2). However, this pattern of findings was not found in females. A non-significant increase in aggression was found in females who received active stimulation compared with those in the sham group, *F* (1, 44) = 1.05, *p* = 0.31. 

### 3.6. Sensitivity Analysis

An intention-to-treat analysis similarly revealed an interaction effect between the stimulation group and gender on aggressive responding in the PSAP, *F* (1, 119) = 4.15, *p* = 0.04, η_p_^2^ = 0.03. Although the effect was attenuated, it remained statistically significant. Furthermore, although it has been suggested that baseline imbalances do not warrant the inclusion of a baseline measure as a covariate [48], additional analysis was conducted to identify whether the observed results were influenced by the imbalance in the age of participants between the stimulation groups. To assess the robustness of the findings, age was included as a covariate in a sensitivity analysis. When age was adjusted for, results were substantively unchanged. The interaction effect between the stimulation group and gender remained statistically significant, *F* (1, 83) = 4.88, *p* = 0.03, η_p_^2^ = 0.06.

## 4. Discussion

This study tests, for the first time, whether an intervention comprising three sessions of anodal tDCS over the prefrontal cortex reduces aggressive behavior in a community sample of adults. Results showed that males who received anodal stimulation over the vmPFC exhibited reduced aggressive responses compared to males in a sham, placebo condition. In females, however, aggressive responses did not significantly differ between the stimulation groups. These findings are bolstered by results from the sensitivity analyses. Despite the null findings, notably, our results are consistent with prior findings obtained for males. In the only previous study that examined the effect of three sessions of prefrontal tDCS on aggression, reductions in aggressiveness were similarly observed in male offenders [26]. This study extends those findings based on self-reports, using a behavioral measure of aggression that, by virtue of having non-aggressive options and associating aggressive responses with a tangible cost, reduces the likelihood of biasing aggressive over-responding. In this way, results from this study provide additional support for the role of the prefrontal cortex in aggression. 

The relationship between increased prefrontal activity and reduction of aggressive behavior may be attributed to various psychological processes. For example, the vmPFC has been repeatedly shown to be critical for affective functions. Emotion regulation and processing may be impaired in individuals with vmPFC deficits to in turn, motivate aggression. Neurological evidence for this supposition comes from patients with vmPFC lesions who show more emotional disturbances [9] and who are suggested not to experience the negative emotions that normally arise in the judgment of attempted harm to another person [55]. Experimental evidence also shows that tDCS delivered to facilitate activity in the vmPFC reduced negative emotions [56]. Another factor that may be involved in the prefrontal-aggression relationship is impulse control as the vmPFC has been implicated in response inhibition [57,58], which is associated with the regulation of aggressive behavior. Although results from the present study revealed that prefrontal stimulation altered aggressive behavior in males to an extent, the mechanisms by which increased prefrontal cortical activity affects aggressive behavior remain to be disentangled in longitudinal tests of mediation. The assessment of an indirect effect would render it possible to identify whether a change in aggression due to stimulation was attributed to a change in a putative mediating variable.

Our findings indicate that there are differences in responsivity to tDCS, which may be attributed to differences in levels of behavioral impairment. Individuals with greater problem behaviors may benefit more from stimulation. This perspective could help explain the gender effects of prefrontal tDCS on aggressive behavior, and why an overall non-significant difference in aggressive responding between the active and sham stimulation groups was found. In this study, males indeed exhibited more aggressive responses compared to females. It has been suggested that an absence of a tDCS effect on aggression in women could be attributed to a floor effect because females, especially in community samples, display lower aggression levels [28]. The notion that prefrontal tDCS may have more pronounced effects on aggression in individuals with existing behavioral impairments is also supported by the finding that anodal tDCS over the right dlPFC inhibited an increase in reactive aggression in alcohol-dependent patients, but not in healthy adults [59]. Gender differences in brain activation have also been documented. For example, compared with females, males were found to exhibit lower empathy-related brain activation when observing another person who played a game unfairly receive physical pain [60]. In the absence of higher levels of aggression, any attempts to increase cortical excitability to help reduce such behavior may be limited, such as in more cognitively-advantaged community-dwelling participants. 

There are some limitations to consider. As participants were only assessed on behavioral aggression on the same day as the last stimulation session, the longer-term effect of tDCS could not be determined. Additionally, the spatial resolution of tDCS is limited. Although the tDCS montage used in this study closely follows that of prior studies stimulating the vmPFC and is guided by a priori software modeling of brain current flow [33], the involvement of other brain areas, such as the dlPFC, cannot be excluded. The lack of a control condition in which the anodal electrode is placed over a comparison brain region precludes firm conclusions about the specificity of the results to the vmPFC. One of the most promising directions for future research is to combine tDCS with brain imaging methods used to measure neural activity. Moreover, despite obtaining open-ended responses from participants about the study objectives at the end of the experimental sessions, the participants were not explicitly asked if they had any doubts about the existence of a real human counterpart in the PSAP, which limits the establishment of the validity of the task. This study also focused on the outcome of reactive aggression. Given that neurobiological correlates of violent behavior may differ between reactive and proactive aggression [61,62], the neural underpinnings of proactive aggression remain to be examined. 

Despite these limitations, this study takes an important step toward addressing some gaps in the extant literature. Recent reviews of studies on non-invasive brain stimulation and aggression have highlighted that there is a need to better understand the effects of different tDCS protocols over different durations [63] and that extant investigations are limited by their generally small sample sizes, with an average of 55 participants in total per study [31]. Furthermore, as empirical support for the association between vmPFC deficits and aggression is not only observed in studies of lesion patients, forensic samples, and violent criminals, but also in samples of healthy subjects, there is a need to shed more light on the brain basis of aggression in healthy adults [7]. Although our present findings need to be replicated, this study helps to address these issues by investigating the utility of multiple sessions of tDCS to modify aggressive behavior in healthy adults. Overall, our results support the notion that individuals from different groups, with different characteristics, may have variable responses to tDCS. In particular, the effects of non-invasive brain stimulation on aggressive behavior may be more strongly expressed in individuals with higher levels of behavioral impairment.

## Figures and Tables

**Table 1:** Comparison of participants who were included and excluded in statistical analyses ^a^.

Characteristic	Included (n = 88)	Excluded (n = 35)	Statistic	*p* Value
*Demographic variables*				
Gender				
Female	46	20	Chi^2^ = 0.24	0.63
Male	42	15		
Age, y	23.82 (5.60)	23.94 (4.73)	t = 0.12	0.91
Ethnicity				
Chinese	72	31	Chi^2^ = 0.84	0.36
Non-Chinese	16	4		
*Baseline measures*				
Aggression	10.16 (4.85)	10.83 (4.55)	t = 0.70	0.48
Psychopathy	55.00 (15.08)	56.09 (13.19)	t = 0.37	0.71
Variety of offending	6.55 (2.91)	7.06 (2.70)	t = 0.90	0.37
Lack of premeditation	1.87 (0.49)	1.84 (0.41)	t = −0.32	0.75
Sensation-seeking	2.91 (0.68)	2.55 (0.66)	t = −2.72	0.01
Cognitive appraisal	31.48 (4.86)	30.34 (5.93)	t = −1.10	0.28
Expressive suppression	17.50 (4.45)	17.23 (4.33)	t = −0.31	0.76
Self-control	40.31 (7.78)	39.69 (7.82)	t = −0.40	0.69
Social adversity	2.00 (1.51)	1.89 (0.93)	t = −0.51	0.61

^a^ Data for continuous variables are presented as mean (SD), with comparisons conducted using independent samples *t*-tests or chi-square tests as appropriate.

**Table 2:** Baseline characteristics by stimulation group and gender ^a^.

Characteristic	tDCS Group (n = 47)	Sham Group (n = 41)	Statistic ^b^	*p* Value	Males (n = 42)	Females (n = 46)	Statistic ^b^	*p* Value
Gender								
Female	22	24	Chi^2^ = 1.21	0.27				
Male	25	17						
Age, y	24.94 (7.35)	22.54 (1.72)	t = −2.17	0.04	24.98 (6.97)	22.76 (3.74)	t = −1.88	0.06
Ethnicity								
Chinese	36	36	Chi^2^ = 1.85	0.17	35	37	Chi^2^ = 0.12	0.73
Non-Chinese	11	5			7	9		
Aggression	10.19 (5.05)	10.12 (4.66)	t = −0.07	0.95	9.95 (5.03)	10.35 (4.72)	t = 0.38	0.71
Psychopathy	55.10 (15.22)	54.88 (15.10)	t = −0.07	0.94	59.74 (15.88)	50.67 (13.03)	t = −2.94	0.004
Variety of offending	6.57 (2.79)	6.51 (3.08)	t = 0.10	0.92	7.24 (3.17)	5.91 (2.53)	t = −2.18	0.03
Lack of premeditation	1.78 (0.50)	1.98 (0.46)	t = 2.01	0.05	1.83 (0.46)	1.91 (0.51)	t = 0.71	0.48
Sensation-seeking	2.92 (0.69)	2.91 (0.67)	t = −0.08	0.94	2.95 (0.60)	2.88 (0.75)	t = −0.50	0.62
Cognitive appraisal	31.09 (5.79)	31.93 (3.52)	t = 0.84	0.41	32.19 (4.96)	30.83 (4.72)	t = −1.32	0.19
Expressive suppression	17.30 (4.39)	17.73 (4.57)	t = 0.45	0.65	18.31 (4.02)	16.76 (4.74)	t = −1.65	0.10
Self-control	41.43 (7.43)	39.02 (8.07)	t = −1.45	0.15	40.67 (8.10)	39.98 (7.56)	t = −0.41	0.68
Social adversity	2.09 (1.63)	1.90 (1.37)	t = −0.56	0.57	2.31 (1.57)	1.72 (1.41)	t = −1.87	0.07

^a^ Data for continuous variables are presented as mean (SD). ^b^ Differences in baseline scores were compared using two-tailed independent *t*-tests and chi-square tests.

**Table 3:** Participant and experimenter conjectures about group assignment and blinding indices after the (**a**) first, (**b**) second, and (**c**) third stimulation sessions.

**(a)**							
	**Participant’s Guess, n**				**James’ BI ^b^**	**Bang’s BI**	**95% CI ^c^**
**Intervention**	tDCS	Sham	Do Not Know	Total			
tDCS	22	10	15	47		0.26	0.07, 0.44
Sham	13	12	15	40		−0.03	−0.23, 0.18
Total	35	22	30	87 ^a^	0.62		0.53, 0.70
	**Experimenter’s Guess, n**						
**Intervention**	tDCS	Sham	Do not know	Total			
tDCS	3	14	30	47		−0.23	−0.37, −0.10
Sham	3	10	28	41		0.17	0.03, 0.31
Total	6	24	58	88	0.84		0.78, 0.89
**(b)**							
	**Participant’s Guess, n**				**James’ BI**	**Bang’s BI**	**95% CI**
**Intervention**	tDCS	Sham	Do not know	Total			
tDCS	31	7	9	47		0.51	0.33, 0.69
Sham	21	9	11	41		−0.29	−0.50, −0.09
Total	52	16	20	88	0.57		0.49, 0.65
	**Experimenter’s Guess, n**						
**Intervention**	tDCS	Sham	Do not know	Total			
tDCS	11	5	30	46		0.13	−0.01, 0.27
Sham	5	8	26	39		0.08	−0.07, 0.23
Total	16	13	56	85 ^a^	0.78		0.70, 0.85
**(c)**							
	**Participant’s Guess, n**				**James’ BI**	**Bang’s BI**	**95% CI**
**Intervention**	tDCS	Sham	Do not know	Total			
tDCS	25	12	10	47		0.28	0.07, 0.48
Sham	20	12	9	41		−0.20	−0.42, 0.03
Total	45	24	19	88	0.59		0.50, 0.67
	**Experimenter’s Guess, n**						
**Intervention**	tDCS	Sham	Do not know	Total			
tDCS	8	14	25	47		−0.13	−0.29, 0.03
Sham	8	12	21	41		0.10	−0.08, 0.28
Total	16	26	46	88	0.77		0.70, 0.84

^a^ 1–3 cases were omitted from calculations of the blinding indices due to missing data. ^b^ BI = blinding index. ^c^ CI = confidence interval.

## Data Availability

The data that support the findings of this study are available on request from the corresponding author, O.C.
